# Anti-obesity effect of Yangkyuksanwha-tang in high-fat diet-induced obese mice

**DOI:** 10.1186/s12906-019-2669-3

**Published:** 2019-09-05

**Authors:** Young-Mee Koh, Soon-Woo Jang, Taek-Won Ahn

**Affiliations:** Department of Sasang Constitutional Medicine, Daejeon University, 4, Notaesan-ro, Seobuk-gu, Cheonan-si, Chungcheongnam-do 331-958 Republic of Korea

**Keywords:** Yangkyuksanwha-tang, Sasang constitutional medicine, Anti-obesity, High-fat diet

## Abstract

**Background:**

Yangkyuksanwha-tang (YST) is an herbal medicine based on Sasang constitutional medicine (SCM) and is widely used in Korean traditional medicine. The aim of the study was to evaluate the effect of YST on obesity in high-fat diet (HFD)-induced obese mice.

**Methods:**

We induced obesity in C57bl/6 J mice using a HFD, and then orally administered 300 mg/kg YST for 6 weeks. We measured body weight, food efficiency, organ and fat weight, serum biochemical parameters, and obesity-related gene expression, and carried out histological analysis at the end of the experimental period.

**Results:**

YST significantly reduced the absolute body weight and food efficiency ratio. The serum, aminotransferase, glucose, total cholesterol, triglyceride, and low-density lipoprotein-cholesterol levels were significantly lower in the YST-treated group than in the control group, whereas the high-density lipoprotein-cholesterol level in the YST-treated group was significantly higher. The YST-treated group also showed a significant reduction in regional fatty tissues and the absolute weight of various organs. We also observed a significantly reduced expression of *AP2/FABP4, C/EBP-β*, leptin, and *SREBP1c/ADD1* mRNA, and significantly increased expression of *UCP-2* and adiponectin mRNA in adipose tissue in the YST-treated group. YST also decreased the lipid droplet size and lipid accumulation in the liver, as well as adipocyte size in epididymal adipose tissue. At the dose tested, YST was non-toxic to the liver and kidneys of the mice.

**Conclusion:**

The results imply that YST has anti-obesity effects in obesity-induced mice. Although the number of experimental animals was limited and the drug effects concern mice, rather than humans, which have different constitutions, the study has valuable implications with respect to the general effects of YST.

## Background

Obesity is a consequence of excess fat accumulation and is a harmful health condition. The World Health Organization standard for being overweight is a body mass index (BMI) above 25 kg/m^2^, and the state of being obese is classified as exceeding a 30 kg/m^2^ BMI. Since 1975, the global population with obesity has almost tripled, with 39% of the adult population diagnosed as overweight and 13% as being obese in 2016 [1]. Intake of high-calorie diets and decrease in physical activities has facilitated the rise in obesity prevalence [2]. Obesity is not only a severe condition in itself, but is also associated with a metabolic syndrome that progresses chronically, which can lead to diabetes, hypertension, and hyperlipidemia [3]. These metabolic syndrome risk factors can potentially lead to cardiovascular disorders, muscular skeletal disease, and cancer [4, 5].

Current treatments for obesity include changes to the diet, psychotherapy for people with eating disorders, and various pharmaceutical interventions. In patients with a BMI higher than 30 kg/m^2^ or with complications, surgical intervention may also be indicated. Commonly used anti-obesity medications include appetite suppressants such as phentermine or diethylpropion, anti-conversant agents such as zonisamide or topiramate, and absorption inhibitors such as orlistat [6]. These drug treatments may carry numerous side effects such as depression, anxiety, headache, dizziness, nausea, and fatigue [7] .

Yangkyuksanwha-tang (YST) is an herbal medicine based on the theory of Sasang constitutional medicine (SCM) and is widely used by Korean oriental medicine practitioners. Sasang constitutional medicine was first postulated in 1894, in the book titled *Donguisusebowon* (東醫壽世保元, Longevity and Life Preservation in Oriental Medicine) [8]. Yangkyuksanwha-tang is widely used in Korean oriental medicine for the treatment of diabetes and obesity. Its effect in reducing blood glucose levels has been demonstrated in experiments with mice [9].

Diabetes is closely related to obesity, as both are associated with insulin resistance and have common complications such as hypertension, hyperlipidemia, and abdominal fat accumulation [10]. Therefore, we hypothesized that YST would also be effective in treating obesity and we sought to verify this through an animal experiment.

YST is composed of eight kinds of herbal plants and one mineral (Table 1). Among the nine constituents of YST, six herbs are known to have lipase inhibitory effects. According to Kim’s research, *Saposhnikovia divaricata* Schischkin, *Schizonepeta tenuifolia* Briquet, *Anemarrhena asphodeloides* Bunge, and *Mentha arvensis* L. var. *piperascens* Malinvaud ex Holmes decreased lipase activity by 91.5, 85.7, 76.0, and 74.8% respectively [11]. *Rehmannia glutinosa* (Gaertner) and *Lonicera japonica* Thunberg are also suggested to be anti-lipase agents in Roh and Jung’s research [12].

**Table 1:** Composition of Yangkyuksanwha-tang

Name	Scientific name	Amount (g)
生地黃	*Rehmannia glutinosa* (Gaertner)	8
忍冬藤	*Lonicera japonica* Thunberg	8
連翹	*Forsythia viridissima* Lindley	8
梔子	*Gardenia jasminoides* Ellis	4
薄荷	*Mentha arvensis* L. var. *piperascens* Malinvaud ex Holmes	4
知母	*Anemarrhena asphodeloides* Bunge	4
石膏	Gypsum (mineral)	4
防風	*Saposhnikovia divaricata* Schischkin	4
荊芥	*Schizonepeta tenuifolia* Briquet	4
Total		48

In addition, previous in vivo experiments show anti-obesity-related effects of the constituents of YST. *Rehmannia glutinosa* (Gaertner) increases the blood levels of high density lipoprotein-cholesterol and insulin in mice [13], and gypsum was reported to decrease the rate of passage of ruminal contents through the gastrointestinal tract via the mechanism of anion-cation imbalance in an in vitro model [14]. Crocetin and crocin, isolated from *Gardenia jasminoides* extract, exert anti-hyperlipidemic effects by inhibiting increases in serum triglycerides and total and low-density lipoprotein (LDL)-cholesterol levels [15]. *Gardenia jasminoides* is also known to have cytoprotective and hepatoprotective activities [16].

Despite its clinical performance in treating obesity and metabolic syndrome, there is still a lack of scientific evidence for the efficacy of YST. Therefore, we aimed to clarify the effectiveness of SCM and YST in treating overweight and obesity in order to develop novel and complementary treatment approaches. In order to test the anti-obesity effect of YST, we used an obesity-induced mouse model and measured body weight, food efficiencies, organ and fat weight, serum biochemistry, obesity-related gene expression, lipid accumulation in the liver, and adipocyte size in the epididymal adipose tissues. The obtained results were compared with those obtained from mice treated with *Garcinia cambogia* extract, which contains hydroxycitric acid (HCA) [17]. The fruit of *Garcinia cambogia* is a popular natural weight loss supplement with no toxic effects [18].

## Methods

### Medicinal plants and mineral

The composition of YST is shown in Table 1. The herbal plants were supplied by Hamsoa Pharmaceutical Co. (Seoul, Republic of Korea). Sensory tests were performed on the samples according to ‘The Korean Herbal Pharmacopoeia’ by KGC Yebon Co. (Director: JaeMyung yoon). Only those that passed herbal Good Manufacturing Practice standards of the Korean Pharmacopoeia were selected and used for this experiment. Voucher specimens have been deposited in the herbarium of the K-Herb Research Center at the Korea Institute of Oriental Medicine. The samples were added to 1 L of distilled water and extracted for 2 h using a boiling pot DWT-1800 T (Daewoong, Korea). The extract was filtered using a 3-mm filter paper (Whatman, Maidstone, England), vacuum evaporated (Evaporator, Eyela, Japan), and freeze dried (Freezedryer, Matsushita, Japan). *Garcinia cambogia* (245 mg/kg; Ethical Naturals, CA. USA) was used as the positive control treatment as its effects are well known, and it is available as an anti-obesity agent.

The dose of YST was determined based on clinical use. In general, a 60 kg adult takes one or two packs of YST in boiled water for a total of 48 g of YST. Considering the conversion factor between mice and humans, a dosage of 300 mg/kg per day of refined YST was considered appropriate for mice [9, 19, 20] .

### Animals and diets

Male, 8-week-old C57bl/6 J mice were purchased from Daehan Biolink Co. (Eumsung, Korea) and maintained for 2 weeks on a commercial diet (AIN-76A diet, Ralston Purina, St. Louis, MO, USA), and water was available ad libitum prior to the experiment. The mice were housed under a 12/12 h dark cycle at 22 ± 2 °C temperature and 50 ± 5% humidity. The experiment was initiated when the mice reached 28–29 g body weight. The mice were then randomly divided into the following four groups of six animals each: normal diet group (C57bl/6 J-Nr), high-fat diet (HFD)-fed control group (HFD-CTL), HFD plus *Garcinia cambogia* extract 245 mg/kg positive control (HFD-GK) group, and HFD plus YST powder 300 mg/kg (HFD-YST) group. The sample size was calculated based on the anti-diabetic and anti-obesity effects of YST according to Lee et al. [19] using G power 3.1. YST and GK were dissolved in a vehicle (0.5% carboxylmethylcellulose) and orally administered once daily for 6 weeks. The experiment was carried out for 6 weeks. The mice were fed a HFD (Rodent Diet D12492; Research Diets, New Brunswick, NJ, USA) consisting of 60% fat, 20% protein, and 20% carbohydrate as an energy resource to induce obesity. The normal diet group mice were fed a commercially available standard chow (Orient Bio Inc., Seongnam, Korea). *Garcinia cambogia* extract was used as the positive control. At the end of the experiment, the mice were fasted for 15 h, anesthetized with ether, 1 mL of blood was sampled directly from the heart of the mice, and then sacrificed. All animal experiments were conducted in accordance with the National Institute of Health guidelines and approved by the Committee on Animal Care of the Daejeon University (Permit No. DJUARB2012–014).

### Measurement of body weight gain and food intake

Body weight gain and food intake were measured using an electronic balance (CATX324, Yangju, Gyeonggi, Korea) at the same time of the day at weekly intervals during the 6-week experimental period. The food efficiency ratio (FER) was calculated as the total weight gain / total food intake × 100.

### Serum assay for biochemical parameters

Fresh whole blood (1 mL) was sampled directly from the heart of the mice into a BD vacutainer tube (CA, USA). Blood samples were centrifuged at 3000 rpm for 15 min at 4 °C, and the samples were stored at − 70 °C. All the serum parameters were measured using an automated biochemical analyzer (Hitachi-720; Hitachi Medical, Tokyo, Japan). The serum levels of leptin and adiponectin were assessed using mouse enzyme-linked immunosorbent assay (ELISA) kits (R & D Systems, Minneapolis, MN, USA), and insulin-like growth factor I (IGF-1) was determined using a similar ELISA kit (Diagnostic Systems Laboratories, Inc., Webster, TX, USA and Linco Research, St Charles, MO, USA).

### Tissue weight and histological analysis

After collecting the blood, subcutaneous, epididymal, kidney, and intestine adipose tissue and the liver, kidney, and spleen were removed and weighed. The tissues were fixed in 10% neutral formalin solution for 24 h and embedded in paraffin for histochemistry. Samples were cut to 6-μm thick sections and stained with hematoxylin and eosin (H & E) or Oil Red O. The size of adipocytes was measured using light microscopy (Olympus BX51; Olympus Optical Co., Tokyo, Japan) and Image-Pro Plus 5.0 software (Medua Cybernetics, Silver Spring, MD, USA).

### Real-time reverse-transcription polymerase chain reaction

TRI Reagent (Sigma-Aldrich) was used to isolate total RNA from the liver and epididymal adipose tissue, and the First Strand cDNA Synthesis Kit (Amersham Pharmacia, Piscataway, NJ, USA) was used to reverse transcribe the total RNA into cDNA. Real-time reverse-transcription polymerase chain reaction (RT-PCR) was carried out using an Applied Biosystems 7500 Real-Time PCR system (Applied Biosystems, Foster City, CA, USA), with probes labeled with 6-carboxy-fluorescein. The PCRs were performed with TaqMan Universal PCR Master Mix containing DNA polymerase (Applied Biosystems). The PCR conditions were as follows: 2 min at 50 °C, 10 min at 95 °C, followed by 40 cycles of 15 s at 95 °C and 1 min at 60 °C. Relative target gene expression was determined by comparing the threshold cycle number at the cross-point between the amplification plot and threshold method with glyceraldehyde 3-phosphate dehydrogenase (GADPH) as the internal control. The expression levels of mRNA were normalized to those of *GAPDH*, and then calculated using the 2^-△△Ct^ method. The primer sequences were as follows: Ap2/FABP4 forward 5′-TGGGAACCTGGAAGCTTGTCTC-3′; Ap2/FABP4 reverse 5′-GAATTCCACGCCCAGTTTGA-3′; C/EBPβ forward 5′-AAGCTGAGCGACGAGTACAAGA-3′; C/EBPβ reverse 5′-GTCAGCTCCAGCACCTTGTG-3′; UCP2 forward 5′-AGTCCCTGCCCTTTGTACACA-3′; UCP2 reverse 5′-GATCCGAGGGCCTCACTAAAC-3′; adiponectin forward 5′-GTCTCAGCTGTCGGTCTTCCCCT-3′; adiponectin reverse 5′-CCCTGGCTTTATGCTCTTTGC-3′; leptin forward 5′-CCAAAACCCTCATCAAGACC-3′; leptin reverse 5′-GTCCAACTGTTGAAGAATGTCCC-3′; SREBP1c forward 5′-AGCCTGGCCATCTGTGAGAA-3′; and SREBP1c reverse 5′-CAGACTGGTACGGGCCACAA-3′.

### Statistical analysis

Data are expressed as mean ± standard error of the mean (SEM). Differences among treatment groups were analyzed by one-way ANOVA and Dunnett’s multiple comparison tests using Prism 7.0 software (GraphPad Software Inc., San Diego, CA). The results with *p*-value ≤0.05 were considered statistically significant.

## Results

### Changes in body weight, food intake, and FER

**Fig. 1:**
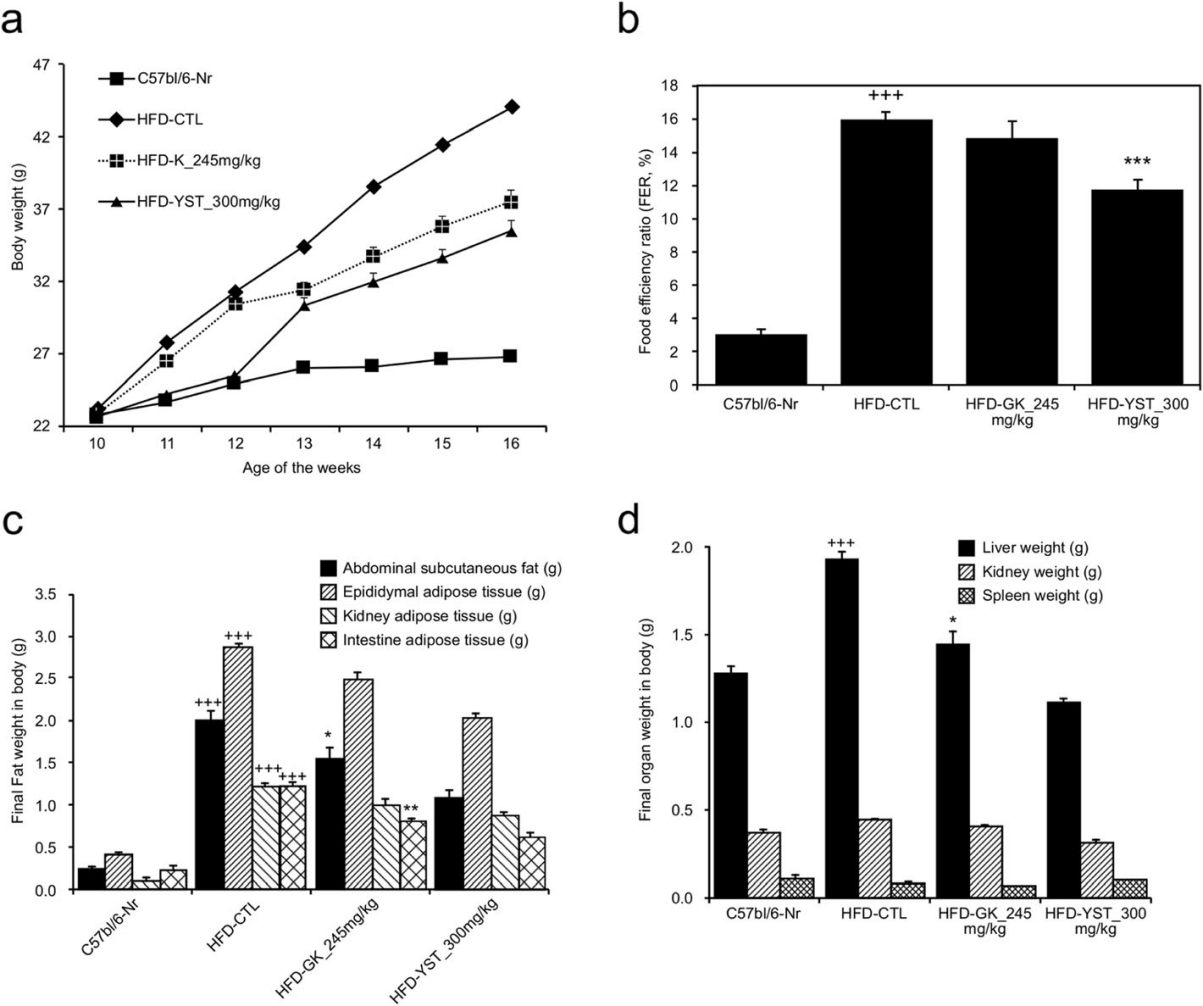
Effects of YST extract in high-fat diet (HFD)-fed mice after 6 weeks of YST administration. **a** Body weight, **b** food efficiency ratio, **c** fat weight, **d** organ weight. Normal diet group: C57bl/6 J-Nr; HFD-control group: HFD-CTL; HFD plus 245 mg/kg *Garcinia cambogia* extract: HFD-GK; HFD plus 300 mg/kg YST: HFD-YST. The results are expressed as mean ± SEM (*n* = 6). +*p* < 0.05, ++*p* < 0.01, and +++*p* < 0.001 compared with C57bl/6 J-Nr, and *p < 0.05, **p < 0.01, and ***p < 0.001 compared with HFD-CTL

We measured absolute body weight as it is a key goal in treating obesity [21]. Food intake was also measured to see changes in appetite, and food efficiency ratio to see changes in metabolism according to intake. Body weight increased by 17.5, 90, 64.4, and 56.38% in the mice that were fed the normal, HFD-CTL, HFD-GK, and HFD-YST diets over the course of the experiment, respectively (Fig. 1, Table 2). The mean body weight at the end of the experiment in the HFD-CTL group was 44.1 ± 2.1 g, which was 64.5% higher than that in the normal group (26.8 ± 1.7 g). Weight gain in the HFD-YST group was 8.6 g less than that in the HFD-CTL group and greater than that in the HFD-GK group, which showed an average of 6.6 g reduction compared with that in the HFD-CTL group (Fig. 1a). The FER of HFD-fed mice was approximately five times higher than that in the normal controls, but was significantly decreased in the HFD-YST group. The diet efficiency rate was 11.73 ± 0.67%, which was significantly different (*p* < 0.001) from that of the control group (HFD-CTL) at 15.97 ± 0.39% (Fig. 1b). These results suggest that YST can inhibit HFD-induced body weight gain.

**Table 2:** Food intake, body weight gain, and food efficiency ratio

Group	Food intake (g/day)	Body weight gain (g/day)	Food efficiency ratio (FER, %)
C57bl/6-Nr	2.71	0.082 ± 0.01	3.02 ± 0.33
HFD-CTL	2.67	0.427 ± 0.01	15.97 ± 0.39 +++
HFD-GK 245 mg/kg	2.01	0.299 ± 0.02	14.89 ± 0.94
HFD-YST 300 mg/kg	2.23	0.262 ± 0.01	11.73 ± 0.67 ***

Normal diet group: C57bl/6 J-Nr; HFD-control group: HFD-CTL; HFD plus 245 mg/kg *Garcinia cambogia* extract: HFD-GK; HFD plus 300 mg/kg YST: HFD-YST. The values are expressed as mean ± SEM (*n* = 6). +*p* < 0.05, ++*p* < 0.01, and +++*p* < 0.001 compared with C57bl/6 J-Nr, and *p < 0.05, **p < 0.01, and ***p < 0.001 compared with HFD-CTL

### Organ and fat weight

Organ and fat weight was measured to see the changes of weight of local parts of the body. The weight of abdominal subcutaneous, epididymal, kidney, and intestinal adipose tissues was increased in the HFD-CTL group, but not in the HFD-YST group (Fig. 1c). The HFD-YST group showed 42.7% lower liver weight, 27.2% lower kidney weight, and 11% lower spleen weight (Fig. 1d) than those of the HFD-CTL group.

### Assessment of liver and kidney function

**Fig. 2:**
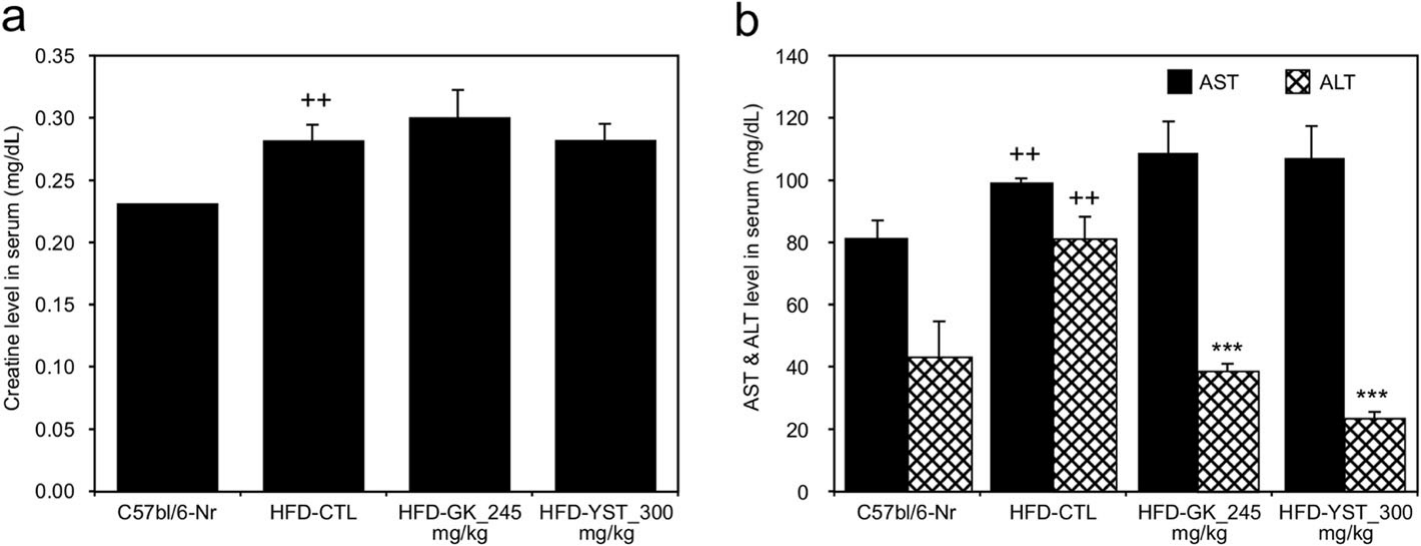
Effects of YST extract on the serum parameters in high-fat diet (HFD)-fed mice. **a** Creatinine, **b** aspartate transaminase (AST) and alanine transaminase (ALT). Normal diet group: C57bl/6 J-Nr; HFD-control group: HFD-CTL; HFD plus 245 mg/kg *Garcinia cambogia* extract: HFD-GK; HFD plus 300 mg/kg YST: HFD-YST. The results are expressed as mean ± SEM (*n* = 6). +*p* < 0.05, ++*p* < 0.01, and +++*p* < 0.001 compared with C57bl/6 J-Nr, and *p < 0.05, **p < 0.01, and ***p < 0.001 compared with HFD-CTL

To evaluate the potential toxicity of YST, the serum alanine aminotransferase (ALT) and aspartate aminotransferase (AST) levels were used as indicators of liver function, and the serum creatinine level was assessed for kidney function. The AST levels of the normal, HFD-CTL, HFD-GK, HFD-YST groups were 80.8, 98.8, 108.8, and 106.8 mg/dL, and the ALT levels were 42.67, 80.5, 38.33, 24.0 mg/dL respectively. Compared with those of the HFD-CTL group, the HFD-YST group did not show a significant increase in the AST level, but showed 70% significant decrease in the ALT level (Fig. 2b). The creatinine levels of the normal, HFD-CTL, HFD-GK, HFD-YST groups were 0.23, 0.28, 0.30, 0.28 mg/dL respectively. The creatinine level in both the HFD-CTL and HFD-YST groups was 0.28 mg/dL and did not show a significant difference (Fig. 2a). The results indicate that YST induces no detectable adverse toxic effects in mice at a dosage of 300 mg/kg for 6 weeks.

### Serum lipid and glucose

The triglycerides, total cholesterol, high-density lipoprotein (HDL)-cholesterol, LDL-cholesterol, and free fatty acid levels were analyzed to see the lipid levels in serum as a result of lipogenesis. We also analyzed glucose level as obesity is the major cause of peripheral insulin resistance and is closely related to the development of altered glucose metabolism. The effects of YST on the serum lipid and glucose levels are shown in Table 3. Both the parameters showed significant results. The serum glucose level in the HFD-YST group was 163.7 mg/dL, which was significantly lower (22.9%) than that (210.5 mg/dL) in the control group (*p* < 0.05). The free fatty acid level in the HFD-YST group was 2.8 mEq/L, lower than that (3.18 mEq/L) in the HFD-CTL group (*p* < 0.001). The total cholesterol and triglyceride levels in the experimental group were 171.8 mg/dL and 76.5 mg/dL, which were significantly lower than those in the HFD-CTL group (*p* < 0.001). The HDL-cholesterol level was 13.2% significantly elevated and the LDL-cholesterol level was 29.5% significantly lowered in the HFD-YST group compared with those in the HFD-CTL group (*p* < 0.001).

**Table 3:** Serum lipid and glucose levels

Group	Normal	HFD-CTL	HFD-GK_245	HFD-YST
Glucose	111.3	210.5 ++	162.2 *	163.7 *
NEFA (Free fatty acid)	2.0	3.2	3.1	2.8 ***
Total cholesterol	107.8	213.8 +++	194.8 ***	171.8 ***
Triglyceride	109.7	172.2 +++	107.2 ***	76.5 ***
HDL-cholesterol	45.2	79.5 +++	81.5	90.0 ***
LDL-cholesterol	14.6	21.0 +++	16.9 ***	14.8 ***

Normal: normal diet group; Control: HFD-negative control group; HFD-GK; HFD-positive control group (*Garcinia cambogia*), HFD-YST: HFD-Yangkyuksanwha-tang administered group (extracts, 300 mg/kg). The values are expressed as mean ± SEM (*n* = 6). +*p* < 0.05, ++*p* < 0.01, and +++*p* < 0.001 compared with ND, and *p < 0.05, **p < 0.01, and ***p < 0.001 compared with HFD-control

### Serum IGF-1, leptin, and adiponectin levels

**Fig. 3:**
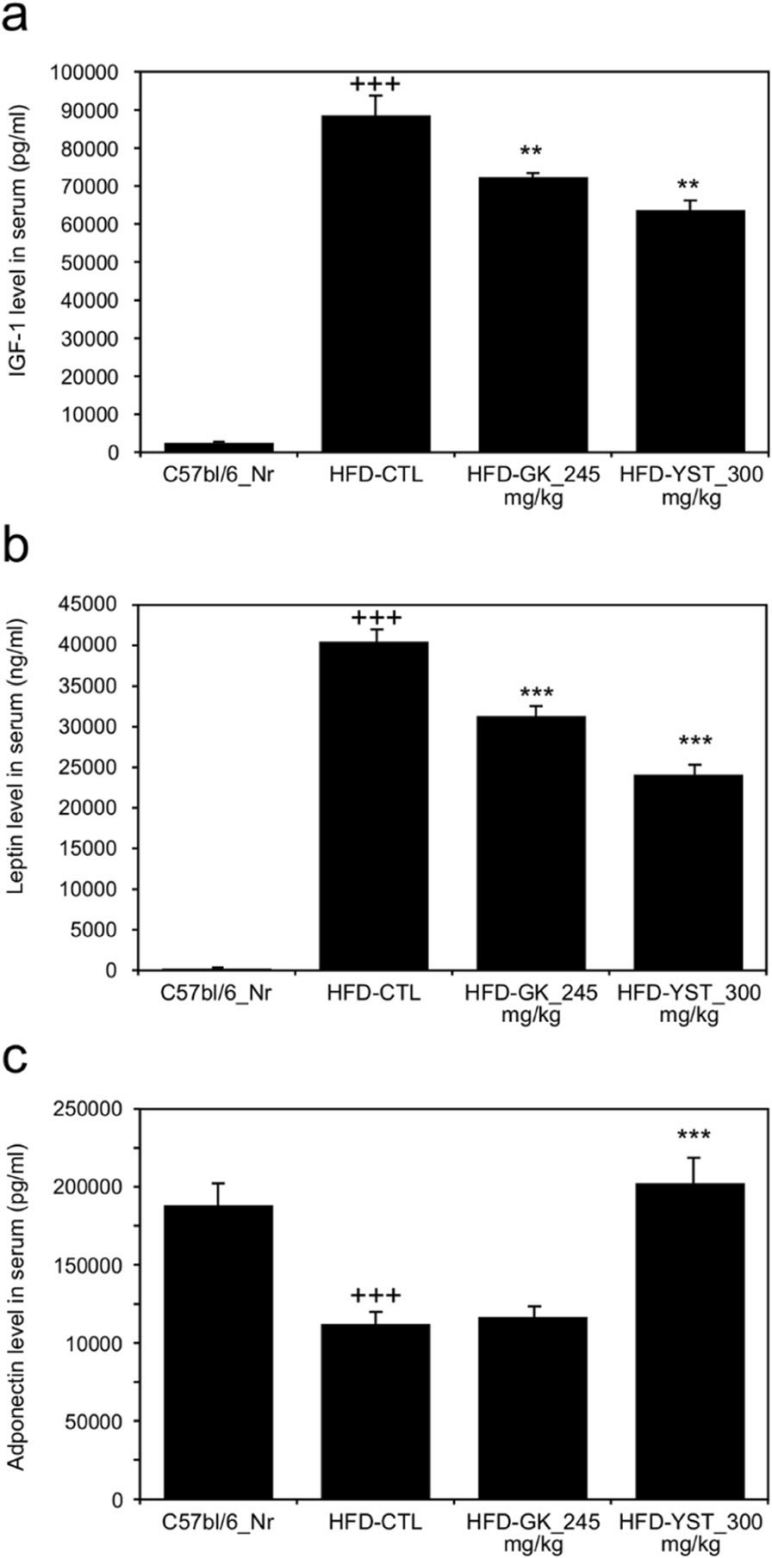
Effects of YST extract on the serum IGF-1, leptin, and adiponectin levels in high-fat diet (HFD)-fed mice, where (**a**) IGF-1, (**b**) leptin, and (**c**) adiponectin. Normal diet group: C57bl/6 J-Nr; HFD-control group: HFD-CTL; HFD plus 245 mg/kg *Garcinia cambogia* extract: HFD-GK; HFD plus 300 mg/kg YST: HFD-YST. The results are expressed as mean ± SEM (*n* = 6). +*p* < 0.05, ++*p* < 0.01, and +++*p* < 0.001 compared with C57bl/6 J-Nr, and *p < 0.05, **p < 0.01, and ***p < 0.001 compared with HFD-CTL

We analyzed serum IGF-1, leptin, and adiponectin levels to see the relationship and its possible role in the development of obesity. The analysis of IGF-1 blood levels of the normal, HFD-CTL, HFD-GK, HFD-YST groups were 2317.45, 88,635.01, 72,483.78, and 63,580.87 pg/ml respectively. The HFD-YST group had 28.2% significantly lower IGF-1 level (*p* < 0.01) than that of the HFD-CTL group (Fig. 3a). The leptin levels of the normal, HFD-CTL, HFD-GK, HFD-YST groups were 243.41, 40,328.21, 31,259.55, and 23,971.99 ng/ml respectively. The HFD-YST group had significantly decreased leptin levels (by 40.5%) compared with that in the HFD-CTL group (*p* < 0.001) (Fig. 3b). The adiponectin levels of the normal, HFD-CTL, HFD-GK, HFD-YST groups were 188,571.24, 111,446.71, 117,557.64, and 202,456.53 pg/ml respectively. The HFD-YST group had significantly higher adiponectin levels (81.6%) than in the HFD-CTL group (*p* < 0.001) (Fig. 3c).

### Expression of lipid metabolism-related genes

**Fig. 4:**
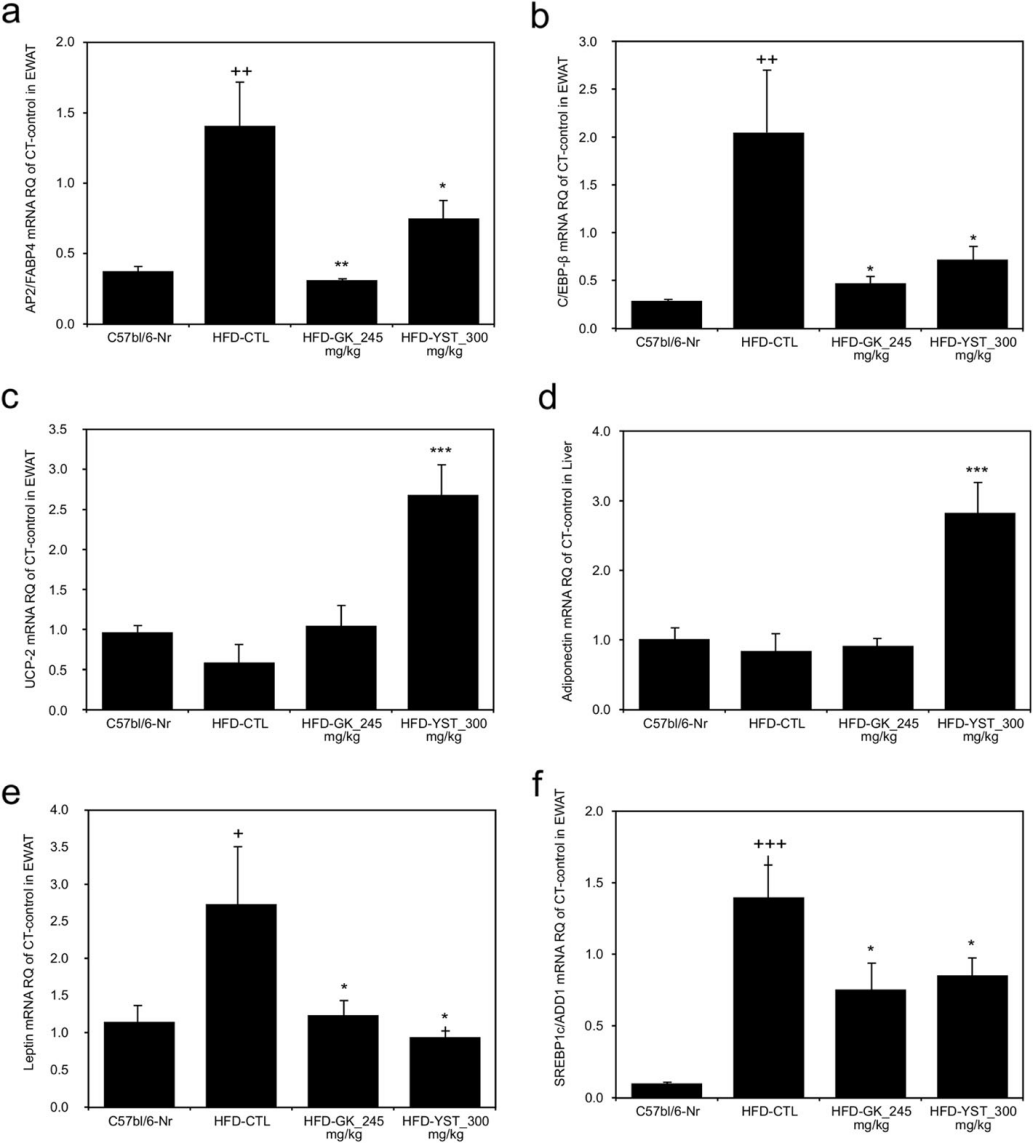
Effects of YST extract on the mRNA expression in epididymal adipose tissue. **a** *AP2/FABP4*, **b** *C/EBP-β,* **c** *UCP-2*, **d** adiponectin, **e** leptin, and **f** *SREBP1c/ADD1*. Normal diet group: C57bl/6 J-Nr; HFD-control group: HFD-CTL; HFD plus 245 mg/kg *Garcinia cambogia* extract: HFD-GK; HFD plus 300 mg/kg YST: HFD-YST. The results are expressed as mean ± SEM (*n* = 6). +*p* < 0.05, ++*p* < 0.01, and +++*p* < 0.001 compared with C57bl/6 J-Nr, and *p < 0.05, **p < 0.01, and ***p < 0.001 compared with HFD-CTL

We analyzed adipogenesis related gene expressions of *AP2/FABP4*, *C/EBP-β*, and *SREBP1c/ADD1*. *UCP-2* mRNA was assessed to see the change in energy consumption. Adiponectin and leptin levels, which serum levels are analyzed, were also assessed genetically. Histological analysis of mRNA in adipose tissue of the liver is shown in Fig. 4. The expression of *AP2/FABP4* mRNA was significantly lower (by 46.7%) in the HFD-YST group than in the HFD-CTL group (*p* < 0.05). Furthermore, the expression of *C/EBP-β* mRNA was significantly higher in the HFD-CTL group and lower (*p* < 0.05) in HFD-YST group than that in the normal group. The *UCP-2* mRNA expression was significantly higher (*p* < 0.001) in the HFD-YST group than in the HFD-CTL and HFD-GK groups. Additionally, the HFD-YST group showed significantly higher (*p* < 0.001) adiponectin mRNA expression and significantly lower (*p* < 0.05) leptin mRNA expression than those in the HFD-CTL. The expression of *SREBP1c/ADD1* mRNA, which influences fat synthesis and transport, was significantly lower (*p* < 0.05) in the HFD-YST group than in the HFD-CTL group.

### Liver histological analysis

**Fig. 5:**
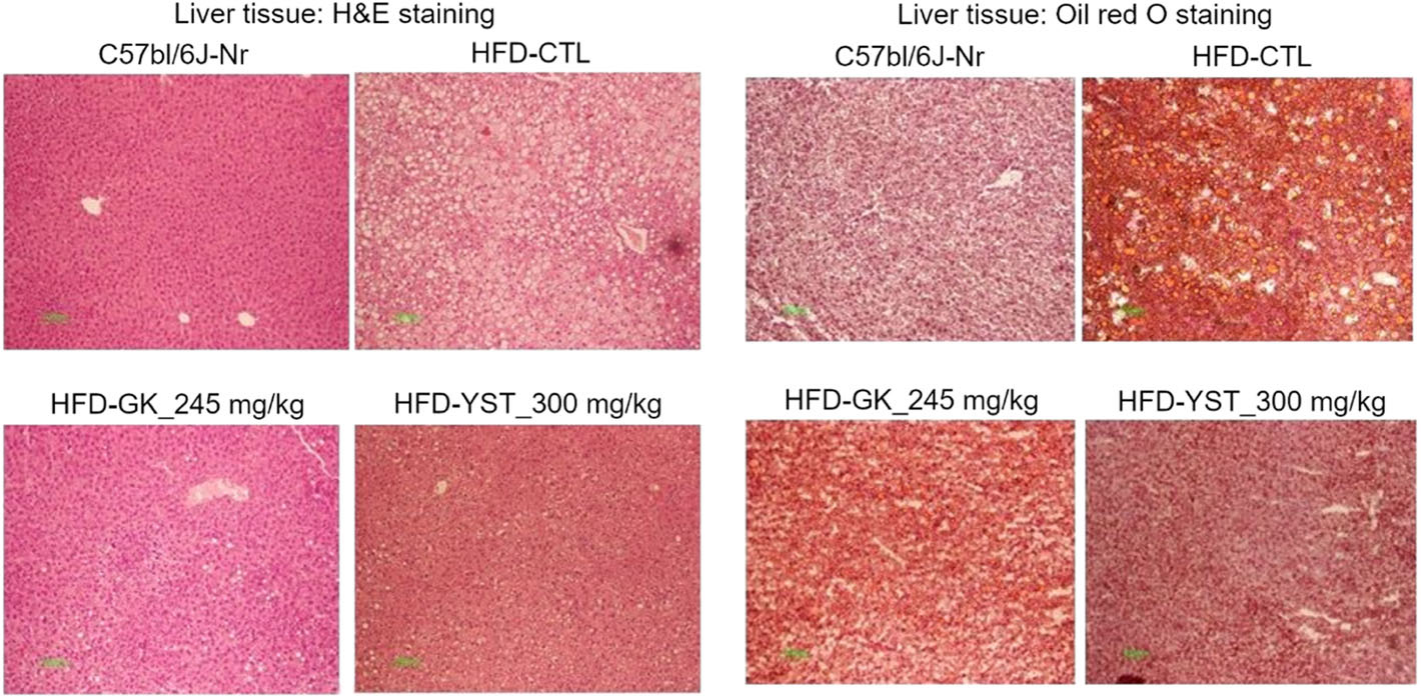
Effect of YST extract on the histological profile of liver in mice that were fed a high-fat diet (HFD) are shown by H&E and Oil red O staining of the liver tissue sections of each group of mice. Normal diet group: C57bl/6 J-Nr; HFD-control group: HFD-CTL; HFD plus 245 mg/kg *Garcinia cambogia* extract: HFD-GK; HFD plus 300 mg/kg YST: HFD-YST

The effect of YST on liver lipid accumulation was analyzed by H&E and Oil Red O staining. The histopathological analysis of the liver tissue showed a significantly larger amount of microscopically identifiable lipid droplets and higher lipid accumulation in the HFD-CTL group than in the normal group (C57bl/6 J-Nr). Meanwhile, the HFD-YST group showed a notable decrease in lipid droplets (Fig. 5).

### Adipocyte size

**Fig. 6:**
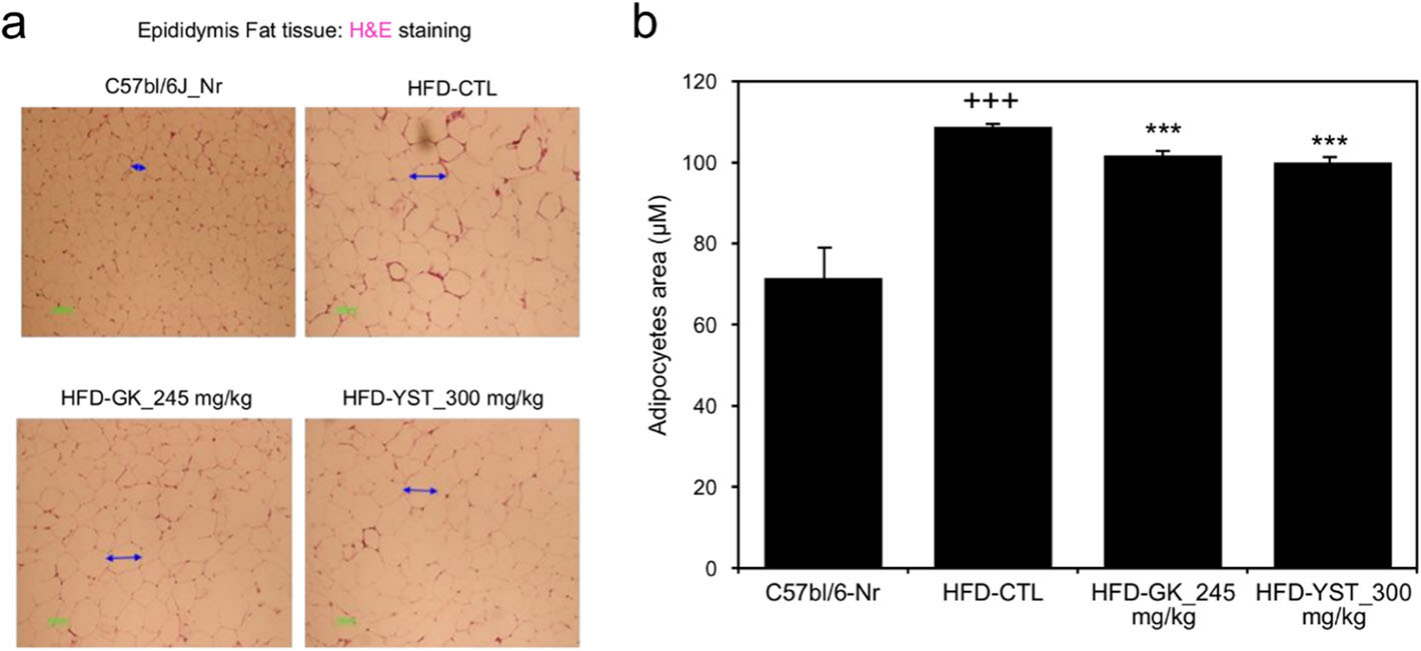
Effects of YST on the histological profile, including (**a**) epididymal adipose tissue morphology and (**b**) adipocyte area, of epididymal adipose tissue in mice that were fed a high-fat diet (HFD). Normal diet group: C57bl/6 J-Nr; HFD-control group: HFD-CTL; HFD plus 245 mg/kg *Garcinia cambogia* extract: HFD-GK; HFD plus 300 mg/kg YST: HFD-YST. The results are expressed as mean ± SEM (*n* = 6). +*p* < 0.05, ++*p* < 0.01, and +++*p* < 0.001 compared with C57bl/6 J-Nr, and *p < 0.05, **p < 0.01, and ***p < 0.001 compared with HFD-CTL

The histological analysis showed larger adipocytes in epididymal adipose tissue of the HFD-CTL group (108.8 μm) than that in the C57bl/6 J-Nr group (71.5 μm). The adipocyte size in the HFD-YST group was 100.0 μm, significantly lower than that in the control group (Fig. 6).

## Discussion

Obesity causes many complications such as metabolic syndrome, type 2 diabetes mellitus, dyslipidemia, cardiovascular diseases, and stroke. These obesity-related diseases are the primary cause of death worldwide [22, 23], leading to the development of various treatments for obesity to prevent its complications. Conventional drugs for obesity have limitations, as they result in various side effects and can only be used by candidates with a BMI over 30 kg/m^2^ or of 27–29.9 kg/m^2^ with comorbidities who are unable to achieve weight loss goals with therapeutic life style changes. Therefore, herbal prescriptions with relatively few side effects that offer the advantage of being useful for various indications have recently been re-investigated [24].

YST was developed to treat those who are overweight or obese, have a strong appetite, prefer cold water, have more bowel movements, sweat more, and dislike heat under normal conditions. In addition, this prescription has been widely used clinically for treating metabolic syndrome and type 2 diabetes mellitus [9]. Despite its clinical usefulness in treating obesity and metabolic syndrome, there is still a lack of scientific evidence regarding its efficacy.

An HFD can be used to induce fatty liver and to increase body weight, visceral adipose tissue, and triglyceride and total cholesterol levels in the serum [25]. An HFD-induced obese mouse model is a critical tool for understanding the interplay of an HFD and the development of obesity [26]. In this study, we evaluated the anti-obesity effects of YST in HFD-induced obese mice. As a result, treatment with YST resulted in anti-obesity effects such as weight loss, decreased FER, suppressed serum lipid and glucose levels, and changes in the expression of lipid metabolism-related genes. Furthermore, YST also improved the histological profiles of the liver and epididymis fat tissues.

Absolute body weight reduction is a key goal in treating obesity, as research has shown that weight loss in overweight or obese adults is associated with a reduction in the mortality rate [21, 27]. In this study, YST treatment was associated with a significant reduction in absolute body weight, food intake, and FER (Table 2, Fig. 1a, b). It has been reported that two major components of YST, *Rehmannia glutinosa* (Gaertner) and *Lonicera japonica* Thunberg, inhibit appetite by reducing levels of plasma ghrelin and inducing peptide YY (PYY) secretion [19]. Moreover, gypsum, one of the main components of YST, was reported to decrease appetite by changing the rate of passage of ruminal contents through the gastrointestinal tract via the mechanism of anion-cation imbalance in an in vitro model [14]. Therefore, the decrease in food intake can be interpreted as a decline in appetite. In addition, the reduction in FER implies that YST either suppresses the absorption of food within the gastrointestinal tract or induces greater metabolic energy expenditure. YST in this study up-regulated the expression of *UCP-2* mRNA, which is translated into mitochondrial uncoupling protein 2, in epididymal white adipose tissues (Fig. 4c). Mitochondrial uncoupling protein 2 increases energy consumption by generating heat instead of ATP in mitochondria via the interruption of oxidative phosphorylation [28]. This is assumed to be one reason for the decrease in FER in the YST group, namely an increase in metabolic energy expenditure through the generation of heat via the up-regulation of *UCP-2* mRNA translation. Thus, inhibition of appetite and a decrease in FER led to a reduction in total weight, and this is the mechanism for the anti-obesity effect of YST.

Lipogenesis, the process by which acetyl-CoA is converted to triglycerides for storage in fat tissues, synthesizes both free fatty acids and triglycerides [29]. The extra free fatty acids and triglycerides go into the liver and are stored as fat tissues [30]. Triglycerides also circulate in the blood, wrapped in a hydrophobic lipoprotein with cholesterol ester. High levels of HDL-cholesterol consistently show an inverse association with coronary events. However, high levels of total cholesterol and all LDL-cholesterol particles increase the risk of atherosclerotic cardiovascular diseases [31, 32]. Therefore, it is important to treat dyslipidemia to prevent cardiovascular disease. In our study, the levels of free fatty acids, triglycerides, total cholesterol, and LDL-cholesterol were reduced in the HFD-YST group compared with that in the positive control group. The YST group showed an improved level of HDL-cholesterol compared with that in the positive group (Table 3). These results indicate that YST may be potentially useful for treating and prevent hyperlipidemia and its complications.

The occurrence of hepatic steatosis without the secondary cause of fat accumulation is called non-alcoholic fatty liver disease (NAFLD) [33]. Excessive HFD feeding can lead to NAFLD, which can cause liver complications such as nonalcoholic steatohepatitis (NASH) and liver cirrhosis [34]. In this study, the HFD-control group presented increased AST and ALT levels in the serum and, histologically, a higher accumulation of lipids in liver tissues than those in the normal group. These results are consistent with those of other studies [35]. Adipose tissue grows by the mechanisms of hypertrophy and hyperplasia [36]. The HFD-control group showed an increased number of larger cells than that in the normal group in the liver and epididymal adipose tissues. However, the HFD-YST group showed a notable decrease in the size of the adipose tissue and the numbers of lipid droplets (Figs. 5 and 6). Lower levels of ALT were also observed in the HFD-YST group compared with that in the HFD-control group (Fig. 2b). These results could be interpreted as the protective effect of YST against liver damage induced by steatohepatitis.

In another regard, AST and ALT levels are widely used biochemical markers for evaluating liver damage, and creatinine levels in the serum are used to assess kidney function. Serum biochemistry results indicated no detectable adverse effects in mice at a dosage of 300 mg/kg for 6 weeks (Fig. 2a, b). This result is consistent with the findings of a previous study, which also reported that YST is safe below a dosage of 5000 mg/kg in mouse models [37].

Type 2 diabetes mellitus is reported to be the result of an impairment in insulin sensitivity and relative insulin deficiency [38, 39]. Type 2 diabetes mellitus is closely related to high serum LDL-cholesterol, low serum HDL-cholesterol, hypertension, and obesity. These features are defined as the metabolic syndrome [40]. In particular, obesity induces peripheral resistance to insulin-mediated glucose uptake. However, this problem can be reversed by weight loss, which restores blood glucose levels to normal [41]. In our study, the serum glucose level was significantly improved in the HFD-YST group compared with that in the HFD-CTL group (Table 3), and the weight gain in the HFD-YST group was 8.6 g less than that in the HFD-CTL group (Fig. 1a), indicating a potential protective effect of YST against glucose tolerance disorders. In other studies, YST and its two major components, *Rehmannia glutinosa* and *Lonicera japonica* Thunberg, resulted in a smaller increase in blood glucose levels and lower levels of HbA1c during long-term treatment in db/db mice [19]. *Rehmannia glutinosa,* in YST was also shown to enhance both basal and glucose-stimulated insulin secretions, as well as the islet insulin content in the pancreatic islets of diabetic mice [13]. These previous findings explain the mechanism by which YST lowers serum glucose levels in this experiment.

Leptin, a 16-kilodalton protein in mouse and human plasma encoded by the obese gene, is known to be closely correlated with BMI and the adipose tissue mass in both mice and humans. Linear correlation was observed between weight loss and the leptin levels in rodents and humans as reduced leptin sensitivity leads to obesity and causes a compensatory increase in plasma leptin levels. So plasma leptin level may be assumed as a marker of body fat increase [42, 43]. To be more specific, leptin exerts its biological effect by increasing the islet volume of the endocrine pancreas to compensate for the impaired function of beta cells in obesity. This characteristic of leptin impairs glucose tolerance and hastens the onset of diabetes in obese individuals. Pancreas-specific leptin receptor-knockout mice actually show improved glucose tolerance compared with a control group [44]. In this study, the HFD-YST group also showed considerably lower serum leptin levels than HFD-CTL and HFD-GK groups (Fig. 3b) and the leptin mRNA expression pattern in the liver was consistent with the serum finding (Fig. 4e).

Adiponectin is a cytokine derived from adipocytes, and it is known to reduce free fatty acids, improve the lipid state, and regulate blood glucose [45]. In particular, lower plasma concentrations of adiponectin are closely associated with insulin resistance and hyperinsulinemia, as well as increasing the risk of type 2 diabetes [46]. Plasma adiponectin level actually shows paradoxical decrease in obese subjects despite of its secretion from adipose tissue [47]. The serum adiponectin level was significantly increased under YST treatment compared with that in the control group (Fig. 3e). The result was consistent with the mRNA level of adiponectin (Fig. 4d). The serum glucose level was significantly improved in the HFD-YST group compared with that in the HFD-CTL group (Table 3). These results suggest that YST improved glucose intolerance and showed anti-obesity effects by regulating the adipocyte-derived hormones leptin and adiponectin at the serum and mRNA levels.

Adipogenesis is regulated by the gene expression of adipocyte protein 2/fatty acid binding protein 4 (AP2/FABP4), CCAAT/enhancer-binding protein β (C/EBP-β), and sterol regulatory element binding protein 1c (SREBP1c/ADD1). When these transcription factors are activated, pre-adipose cells are differentiated into mature fat cells [48–50]. The expression profiles of AP2/FABP4 and C/EBP-β were favorable under YST treatment, suggesting that YST simultaneously suppresses adipogenesis (Fig. 4a, b, f). C/EBP-β is induced during the early stages of adipogenesis and has a decisive role in the completion of adipocyte differentiation. In a previous in vivo experiment, the expression of C/EBP-α and PPARγ alone without C/EBP-β and C/EBP-δ did not result in completion of adipogenesis [51]. In this study, the YST group showed a lower expression of *C/EBP-β* mRNA (Fig. 4b). In addition, according to Shin’s study, YST treatment down-regulated peroxisome proliferator-activated receptor-gamma (PPARγ) at the mRNA level in adipocytes. And this effect substantially reduced the number of lipid droplets and content of triglyceride in the adipose tissue of mice 3 T3-L1 cells [52]. In our study, the HFD-YST group also showed significantly reduced adipocyte size compared with the HFD-CTL group (Fig. 6a, b).

Lipogenesis is highly regulated by sterol regulatory element binding proteins (SREBPs) [53]. SREBPs are a family of transcription factors that participate in lipogenesis by controlling the expression of a range of enzymes required for endogenous cholesterol, fatty acid, and triacylglycerol synthesis. In vivo studies using transgenic and knockout mice suggest that SREBP1c/ADD1 is involved in free fatty acid synthesis and insulin-induced glucose metabolism [49]. In our study, there was a lower expression of *SREBP1c/ADD1* mRNA in the YST group (Fig. 4f). These results suggest that YST affects the expression of obesity-related transcription factors in white adipose tissues. Changes in the expression of obesity-related genes are considered to be the mechanism by which YST improves free fatty acid, total cholesterol, triglyceride, and LDL-cholesterol profiles and leads to reduced fat weight and adipose tissue (Fig. 1c, d). The above results showed anti-obesity effects of YST by regulating adipogenesis and lipogenesis through the changes in the expression of obesity-related transcription factors at mRNA level.

In summary, YST, which is the combined prescription of eight herbs and one mineral, appears to exert a considerable anti-obesity effect via a number of pathways. We observed regulation of appetite and changes in the expression of obesity-related transcription factors and adipocyte-related hormones in an HFD-induced obese mouse model. Additional studies are needed to further investigate the mechanisms of the anti-obesity effect of YST in greater detail. Clinical trials in humans with obesity are anticipated.

## Conclusions

In conclusion, YST treatment reduced the absolute body weight, organ fat weight, and FER (Food Efficiency Ratio) of HFD-induced obese mice by regulating obesity-related transcription factors and adipocyte-derived hormones. YST also ameliorated serum glucose levels and lipid profiles in the serum. These results elucidate the effects of YST and validate traditional knowledge. Thus, the use of YST is considered to be a supplementary treatment for obesity together with conventional treatments.

## Data Availability

All data generated or analyzed during this study are included in this published article.

## Abbreviations

ALT: Alanine aminotransferase
AST: Aspartatae aminotransferase
BMI: Body mass index
ELISA: Enzyme-linked immunosorbent assay
FER: Food efficiency ratio
GADPH: Glyceraldehyde 3-phosphate dehydrogenase
HDL: High-density lipoprotein
HFD: High-fat diet
HFD-CTL: High-fat diet-fed control group
HFD-GK: HFD plus *Garcinia cambogia* extract 245 mg/kg positive control
HFD-YST: HFD plus YST powder 300 mg/kg
IGF-1: Insulin-like growth factor I
LDL: Low-density lipoprotein
RT-PCR: Real-time reverse-transcription polymerase chain reaction
SCM: Sasang constitutional medicine
SEM: Standard error of the mean
YST: Yangkyuksanwha-tang

## References

[1] World Health Organization (2018). Obesity and overweight fact sheet.

[2] Lebel A, Kestens Y, Pampalon R, Theriault M, Daniel M, Subramanian SV (2012). Local context influence, activity space, and foodscape exposure in two Canadian metropolitan settings: is daily mobility exposure associated with overweight?. J Obes.

[3] Guh DP, Zhang W, Bansback N, Amarsi Z, Birmingham CL, Anis AH (2009). The incidence of co-morbidities related to obesity and overweight: a systematic review and meta-analysis. BMC Public Health.

[4] Lenz M, Richter T, Muhlhauser I (2009). The morbidity and mortality associated with overweight and obesity in adulthood: a systematic review. Dtsch Arztebl Int.

[5] Oh SW, Yoon YS, Shin SA (2005). Effects of excess weight on cancer incidences depending on cancer sites and histologic findings among men: Korea National Health Insurance Corporation Study. J Clin Oncol.

[6] Li Z, Maglione M, Tu W, Mojica W, Arterburn D, Shugarman LR (2005). Meta-analysis: pharmacologic treatment of obesity. Ann Intern Med.

[7] Kang JG, Park CY (2012). Anti-obesity drugs: a review about their effects and safety. Diabetes Metab J.

[8] Kim JY, Pham DD (2009). Sasang constitutional medicine as a holistic tailored medicine. Evid Based Complement Alternat Med.

[9] Shin SW, Lee S-G, Jang H-J, Ahn KS, Lee E, Koh B-H (2014). Review of experimental and clinical studies on Soyangin (少陽人) Yanggyeoksanhwa-tang (凉膈散火湯) in Korea since 2000. Orient Pharm Exp Med.

[10] Isomaa B, Almgren P, Tuomi T, Forsen B, Lahti K, Nissen M (2001). Cardiovascular morbidity and mortality associated with the metabolic syndrome. Diabetes Care.

[11] Kim JH, Kim KJ (2005). Experimental study on anti-obesity effect according to inhibitory effect against lipase activity of sasang constitution medicines. J Physiol Pathol Korean Med.

[12] Roh C, Jung U (2012). Screening of crude plant extracts with anti-obesity activity. Int J Mol Sci.

[13] Zhou J, Xu G, Yan J, Li K, Bai Z, Cheng W (2015). *Rehmannia glutinosa* (Gaertn.) DC. Polysaccharide ameliorates hyperglycemia, hyperlipemia and vascular inflammation in streptozotocin-induced diabetic mice. J Ethnopharmacol.

[14] Kroger D, Carroll FD (1964). Possible mechanism of agricultural gypsum in regulating appetite. J Anim Sci.

[15] Lee IA, Lee JH, Baek NI, Kim DH (2005). Antihyperlipidemic effect of crocin isolated from the fructus of Gardenia jasminoides and its metabolite Crocetin. Biol Pharm Bull.

[16] Lee SJ, Oh PS, Lim KT (2006). Hepatoprotective and hypolipidaemic effects of glycoprotein isolated from *Gardenia jasminoides* Ellis in mice. Clin Exp Pharmacol Physiol.

[17] Heymsfield SB, Allison DB, Vasselli JR, Pietrobelli A, Greenfield D, Nunez C (1998). *Garcinia cambogia* (hydroxycitric acid) as a potential antiobesity agent: a randomized controlled trial. JAMA.

[18] Fassina P, Scherer Adami F, Terezinha Zani V, Kasper Machado IC, Garavaglia J, Quevedo Grave MT (2015). The effect of *Garcinia cambogia* as coadjuvant in the weight loss process. Nutr Hosp.

[19] Lee IS, Kim KS, Kim KH, Park J, Jeong HS, Kim Y (2016). Anti-diabetic and anti-obesitic effects of aqueous extracts of Yangkyuksanhwa-tang and its two major compositions on db/db mice. Biomed Pharmacother.

[20] Nair AB, Jacob S (2016). A simple practice guide for dose conversion between animals and human. J Basic Clin Pharm.

[21] Poobalan AS, Aucott LS, Smith WCS, Avenell A, Jung R, Broom J (2007). Long-term weight loss effects on all cause mortality in overweight/obese populations. Obes Rev.

[22] Knowler WC, Barrett-Connor E, Fowler SE, Hamman RF, Lachin JM, Walker EA (2002). Reduction in the incidence of type 2 diabetes with lifestyle intervention or metformin. N Engl J Med.

[23] Aune D, Sen A, Norat T, Janszky I, Romundstad P, Tonstad S (2016). Body mass index, abdominal fatness, and heart failure incidence and mortality: a systematic review and dose-response meta-analysis of prospective studies. Circulation.

[24] Calixto JB (2000). Efficacy, safety, quality control, marketing and regulatory guidelines for herbal medicines (phytotherapeutic agents). Braz J Med Biol Res.

[25] Hariri N, Thibault L (2010). High-fat diet-induced obesity in animal models. Nutr Res Rev.

[26] Wang CY, Liao JK (2012). A mouse model of diet-induced obesity and insulin resistance. Methods Mol Biol.

[27] Sjostrom L (2013). Review of the key results from the Swedish obese subjects (SOS) trial - a prospective controlled intervention study of bariatric surgery. J Intern Med.

[28] Boss O, Samec S, Desplanches D, Mayet MH, Seydoux J, Muzzin P (1998). Effect of endurance training on mRNA expression of uncoupling proteins 1, 2, and 3 in the rat. FASEB J.

[29] Kersten S (2001). Mechanisms of nutritional and hormonal regulation of lipogenesis. EMBO Rep.

[30] Bederman IR, Foy S, Chandramouli V, Alexander JC, Previs SF (2009). Triglyceride synthesis in epididymal adipose tissue: contribution of glucose and non-glucose carbon sources. J Biol Chem.

[31] Parish S, Offer A, Clarke R, Hopewell JC, Hill MR, Otvos JD (2012). Lipids and lipoproteins and risk of different vascular events in the MRC/BHF heart protection study. Circulation.

[32] Mora S, Buring JE, Ridker PM, Cui Y (2011). Association of high-density lipoprotein cholesterol with incident cardiovascular events in women, by low-density lipoprotein cholesterol and apolipoprotein B100 levels: a cohort study. Ann Intern Med.

[33] Caldwell SH, Oelsner DH, Iezzoni JC, Hespenheide EE, Battle EH, Driscoll CJ (1999). Cryptogenic cirrhosis: clinical characterization and risk factors for underlying disease. Hepatology.

[34] Nakamura A, Terauchi Y (2013). Lessons from mouse models of high-fat diet-induced NAFLD. Int J Mol Sci.

[35] Jang JW, Lim DW, Chang JU, Kim JE (2018). The combination of *Ephedrae herba* and *Coicis semen* in Gambihwan attenuates obesity and metabolic syndrome in high-fat diet-induced obese mice. Evid Based Complement Alternat Med.

[36] Jo J, Gavrilova O, Pack S, Jou W, Mullen S, Sumner AE (2009). Hypertrophy and/or hyperplasia: dynamics of adipose tissue growth. PLoS Comput Biol.

[37] Ma J-Y, Huang D-S, Seo C-S, Lee S-W, Kim J-Y, Shin H-K (2009). Acute toxicity study on Yangkyuksanhwa-tang in mice. J Sasang Constitut Med.

[38] Beck-Nielsen H, Groop LC (1994). Metabolic and genetic characterization of prediabetic states. Sequence of events leading to non-insulin-dependent diabetes mellitus. J Clin Invest.

[39] Kahn CR (1994). Banting lecture. Insulin action, diabetogenes, and the cause of type II diabetes. Diabetes.

[40] DeFronzo RA (1997). Insulin resistance: a multifaceted syndrome responsible for NIDDM, obesity, hypertension, dyslipidaemia and atherosclerosis. Neth J Med.

[41] Friedman JE, Dohm GL, Leggett-Frazier N, Elton CW, Tapscott EB, Pories WP (1992). Restoration of insulin responsiveness in skeletal muscle of morbidly obese patients after weight loss. Effect on muscle glucose transport and glucose transporter GLUT4. J Clin Invest.

[42] Halaas JL, Gajiwala KS, Maffei M, Cohen SL, Chait BT, Rabinowitz D, Lallone RL, Burley SK, Friedman JM (1995). Weight-reducing effects of the plasma protein encoded by the obese gene. Science.

[43] Maffei M, Halaas J, Ravussin E, Pratley RE, Lee GH, Zhang Y, Fei H, Kim S, Lallone R, Ranganathan S (1995). Leptin levels in human and rodent: measurement of plasma leptin and Ob RNA in obese and weight-reduced subjects. Nat Med.

[44] Morioka T, Asilmaz E, Hu J, Dishinger JF, Kurpad AJ, Elias CF (2007). Disruption of leptin receptor expression in the pancreas directly affects beta cell growth and function in mice. J Clin Invest.

[45] Meier U, Gressner AM (2004). Endocrine regulation of energy metabolism: review of pathobiochemical and clinical chemical aspects of leptin, ghrelin, adiponectin, and resistin. Clin Chem.

[46] Li S, Shin HJ, Ding EL, van Dam RM (2009). Adiponectin levels and risk of type 2 diabetes: a systematic review and meta-analysis. JAMA.

[47] Arita Y, Kihara S, Ouchi N, Takahashi M, Maeda K, Miyagawa J, Hotta K, Shimomura I, Nakamura T, Miyaoka K (1999). Paradoxical decrease of an adipose-specific protein, adiponectin, in obesity. Biochem Biophys Res Commun.

[48] Gregoire FM, Smas CM, Sul HS (1998). Understanding adipocyte differentiation. Physiol Rev.

[49] Eberle D, Hegarty B, Bossard P, Ferre P, Foufelle F (2004). SREBP transcription factors: master regulators of lipid homeostasis. Biochimie.

[50] Rosen ED, Walkey CJ, Puigserver P, Spiegelman BM (2000). Transcriptional regulation of adipogenesis. Genes Dev.

[51] Tanaka T, Yoshida N, Kishimoto T, Akira S (1997). Defective adipocyte differentiation in mice lacking the C/EBPbeta and/or C/EBPdelta gene. EMBO J.

[52] Jeong SJ, Yoo SR, Seo CS, Shin HK (2015). Traditional medicine yanggyuksanhwa-tang inhibits adipogenesis and suppresses proliferator-activated receptor-gamma expression in 3T3-L1 cells. Pharmacogn Mag.

[53] Hua X, Yokoyama C, Wu J, Briggs MR, Brown MS, Goldstein JL (1993). SREBP-2, a second basic-helix-loop-helix-leucine zipper protein that stimulates transcription by binding to a sterol regulatory element. Proc Natl Acad Sci U S A.

